# Dynamic functional modules in co-expressed protein interaction networks of dilated cardiomyopathy

**DOI:** 10.1186/1752-0509-4-138

**Published:** 2010-10-15

**Authors:** Chen-Ching Lin, Jen-Tsung Hsiang, Chia-Yi Wu, Yen-Jen Oyang, Hsueh-Fen Juan, Hsuan-Cheng Huang

**Affiliations:** 1 Institute of Biomedical Informatics, Center for Systems and Synthetic Biology, National Yang-Ming University, Taipei 112, Taiwan; 2 Graduate Institute of Biomedical Electronics and Bioinformatics, Center for Systems Biology and Bioinformatics, National Taiwan University, Taipei 106, Taiwan; 3 Department of Physics, National Dong Hwa University, Hualien 974, Taiwan; 4 Department of Life Science, Institute of Molecular and Cellular Biology, National Taiwan University, Taipei 106, Taiwan

## Abstract

**Background:**

Molecular networks represent the backbone of molecular activity within cells and provide opportunities for understanding the mechanism of diseases. While protein-protein interaction data constitute static network maps, integration of condition-specific co-expression information provides clues to the dynamic features of these networks. Dilated cardiomyopathy is a leading cause of heart failure. Although previous studies have identified putative biomarkers or therapeutic targets for heart failure, the underlying molecular mechanism of dilated cardiomyopathy remains unclear.

**Results:**

We developed a network-based comparative analysis approach that integrates protein-protein interactions with gene expression profiles and biological function annotations to reveal dynamic functional modules under different biological states. We found that hub proteins in condition-specific co-expressed protein interaction networks tended to be differentially expressed between biological states. Applying this method to a cohort of heart failure patients, we identified two functional modules that significantly emerged from the interaction networks. The dynamics of these modules between normal and disease states further suggest a potential molecular model of dilated cardiomyopathy.

**Conclusions:**

We propose a novel framework to analyze the interaction networks in different biological states. It successfully reveals network modules closely related to heart failure; more importantly, these network dynamics provide new insights into the cause of dilated cardiomyopathy. The revealed molecular modules might be used as potential drug targets and provide new directions for heart failure therapy.

## Background

Protein-protein interactions (PPI) are of central importance for most biological processes, and thus the protein interaction network (PIN) provides a global picture of cellular mechanisms. With the accumulation of interactome and transcriptome data, the integration of gene expression profiles has revealed the dynamics of protein interaction networks. For example, *Han et al*. [1] analyzed the protein interaction network of yeast and uncovered two types of hub proteins: "party" hubs and "date" hubs, which displayed condition- or location-specific features in the interactome network. *Xue et al*. [2] developed a new analytic method to discover the dynamic modular structure of the human protein interaction network in their aging study. Recently, *Taylor et al*. [3] also proposed another two types of hub proteins: intermodular hubs and intramodular hubs, and identified whether the interactions between proteins were context specific or constitutive in the human protein interaction network. Similar techniques were also applied to reveal disease related genes or modules. *Chuang et al*. [4] improved the prognostic predictive performance of gene expression signatures by incorporating interactome data in breast cancer. *Taylor et al*. [3] used a method analogous to a previous study [1] and revealed that dynamic modularity of the human protein interaction network may be a good indicator of breast cancer prognosis. In the context of heart failure, *Camargo *and *Azuaje *[5,6] integrated gene expression profiles with the protein interaction network in human dilated cardiomyopathy and efficiently identified potential novel DCM signature genes and drug targets. These studies suggest that the integration of interactome data with transcriptome information may facilitate the identification or discovery of disease biomarkers.

Heart failure is one of the main causes of death in the world and is the consequence of many complex factors including genetics, diet, environment and lifestyle. Heart failure is a physiological state in which the heart cannot provide sufficient output of blood to meet the body's needs. Dilated cardiomyopathy (DCM), the major cause of heart failure, impairs the blood pumping ability of the heart and leads to insufficient blood flow to vital organs due to the enlargement and weakening of the heart [7]. Previous studies [8-10] on gene expression profiles have provided distinct perspectives on the etiology of DCM. *Barth et al*. [8] pointed out the significant immune response processes involved in end-stage DCM and presented a robust gene expression signature of this disease. *Wittchen et al*. [10] suggested novel therapeutic targets by gene expression profile analysis of human inflammatory cardiomyopathy. *Kabb et al*. [9] analyzed microarray dataset of human myocardial tissue to obtain region- and DCM-specific transcription profiles and determined the gene expression fingerprint of DCM. Even though various causes of DCM have been revealed, the underlying molecular mechanism of this disease remains unclear.

Here, we developed a network-based analysis approach to discover DCM or non-DCM related functional subnetworks by integrating DCM related gene expression profiles with the human protein interaction network and Gene Ontology (GO) annotations. A comparative analysis was utilized to extract DCM exclusive subnetworks as heart failure related modules. These modules could be used to classify normal and disease samples. We further investigated the co-expressed protein interaction network structures of each module for DCM and non-DCM and observed dynamic variations of the identified modules between the two states. Our results suggest that the modular changes between DCM and non-DCM could imply plausible molecular mechanisms involved in heart failure progression.

## Results

### Condition-Specific Co-expressed Protein Interaction Networks

We integrated gene expression profiles with the protein interaction network to construct condition-specific co-expressed protein interaction networks of DCM and non-DCM, respectively. Two interacting proteins are called co-expressed protein-protein interaction (CePPI) under a specified condition, if their encoding genes are expressed correlatively with each other under particular biological conditions, such as DCM or non-DCM in this study. The network that consists of all the CePPIs under the DCM condition is abbreviated as DCM CePIN and the network under the non-DCM condition as non-DCM CePIN. Table 1 summarizes the structural information of DCM and non-DCM networks. Given that the gene expression profiles were derived from the same tissue [8], DCM and non-DCM CePINs shared almost 70% of proteins and nearly 80% of physical protein interactions in common. However, the shared CePPIs were only approximately 30%, indicating a large amount of interaction rewiring between DCM and non-DCM. Hence, CePPIs may reveal the condition-specific dynamic information hidden among otherwise common static interactions. Previous studies [1,11] have shown that by integrating co-expression information into physical protein interaction networks, dynamic features among various networks can be extracted. This preliminary observation of the network structure indicates the limitation of a static protein interaction network, and demonstrates the necessity of applying a condition-specific co-expressed protein interaction network model to discover the dynamic features which are attributed to the changes in gene expression profiles inside the static biological networks.

**Table 1:** Structural information of DCM and non-DCM networks

	Proteins	PPIs	Co-expressed PPIs
DCM	3160	13928	4306
non-DCM	3302	14557	4872
Overlap	2226	11092	1425

### Hub Proteins in CePINs Tend to Be Differentially Expressed

**Figure 1:**
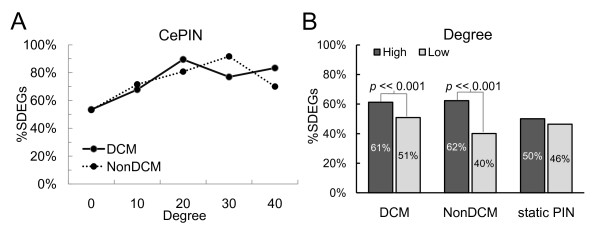
**Correlations between CePPI degree and SDEG proportion**. (A) The line chart shows that the proportion tendencies of SDEGs in terms of degree are positively correlated in DCM and non-DCM CePINs. (B) The bar chart displays the SDEG proportions for proteins with high- (top 50%) and low-degree (last 50%) in each network; DCM CePIN, non-DCM CePIN and static PIN. Both DCM and non-DCM CePINs represent significant differences of SDEG proportions between high- and low-degree proteins. The results indicate that the SDEG proportions are positively correlated with the CePIN degree rather than the static PIN.

To further investigate the features of condition-specific CePINs, we examined their topological properties, including degree, betweenness centrality, closeness centrality and clustering coefficient for significantly differentially expressed genes (SDEGs). All four topological properties of SDEGs were significantly higher than those of non-SDEGs in DCM CePIN, as well as non-SDEGs in non-DCM CePIN (Table 2). The results showed that the proteins encoded by differentially expressed genes under a specific biological condition tended to play topologically important roles in the corresponding CePINs. Figure 1A shows that proteins with higher CePPI degrees have larger proportions of SDEGs. However, due to the fact that hub proteins in static PIN had higher CePPI degrees in condition-specific CePINs, we examined high- and low-degree proteins in three types of networks respectively, and compared their proportions of SDEGs (Figure 1B). Although the proportion of SDEGs (50%) for high-degree proteins in static PIN was similar to those with a low-degree (46%), SDEGs were especially enriched for high-degree proteins in both DCM CePIN and non-DCM CePIN (> 60%) in comparison with low-degree proteins (Fisher's exact test, *P *< 0.001). The results implied that hub proteins in CePINs are more likely to be differentially expressed, suggesting modular rewiring of co-expressed interactions between different biological states. Altogether, the condition-specific CePINs could carry more biologically significant network structure that reflects the underlying gene expression dynamics.

**Table 2:** Comparison of key topological properties

		DCM CePIN	
Property	SDEGs	Non-SDEGs	*P*-value
Degree	3.47	2.51	9.58E-09
Betweenness centrality	0.0023	0.0012	1.79E-07
Closeness centrality	0.1769	0.1734	4.50E-03
Clustering coefficient	0.04	0.03	2.23E-04
		Non-DCM CePIN	
Degree	3.72	2.6	7.64E-15
Betweenness centrality	0.0021	0.0011	8.79E-15
Closeness centrality	0.1837	0.1796	1.64E-04
Clustering coefficient	0.04	0.03	1.81E-07

The average of each topological property for SDEGs and non-SDEGs in either DCM or non-DCM CePIN is given in the table together with the corresponding *P*-value. The *P*-values were calculated using non-parametric Wilcoxon rank-sum tests.

Hub proteins of DCM CePIN were also studied in the context of Gene Ontology to identify major biological roles in each protein category. Table 3 shows the top 10 significantly over-represented functional annotations of DCM hub proteins in GO biological process category (level 6) by BiNGO [12] (Hypergeometric test, Benjamini & Hochberg FDR correction, *P *≤ 0.01). Previously, Camargo and colleagues [6] analyzed the differentially expressed genes of DCM in the context of GO and identified phosphorylation to be significantly over-represented, which is also the most significant functional annotation of hub proteins in DCM CePIN from our study. Kang and colleagues [13] suggested that DCM is highly associated with dysfunction of apoptosis pathways. Consistent with their study, apoptosis-related functions, including regulation of apoptosis, apoptosis, and positive regulation of programmed cell death, came up in our GO identification. These findings were consistent with what has been observed in previous studies and imply that hub proteins in CePINs are similar to SDEGs with respect of their biological roles.

**Table 3:** Top 10 significant level-6 GO annotations of hubs

GO term	Description	*P*-value
16310	**phosphorylation**	2.40E-12
7167	enzyme linked receptor protein signaling pathway	3.01E-10
6464	protein modification process	7.28E-08
42981	**regulation of apoptosis**	1.41E-05
6915	**apoptosis**	2.09E-05
31325	positive regulation of cellular metabolic process	2.79E-05
43068	**positive regulation of programmed cell death**	4.35E-05
48340	paraxial mesoderm morphogenesis	4.95E-05
48339	paraxial mesoderm development	4.95E-05
45941	positive regulation of transcription	9.67E-05

### Identification of Two DCM-Related Functional Modules

**Figure 2:**
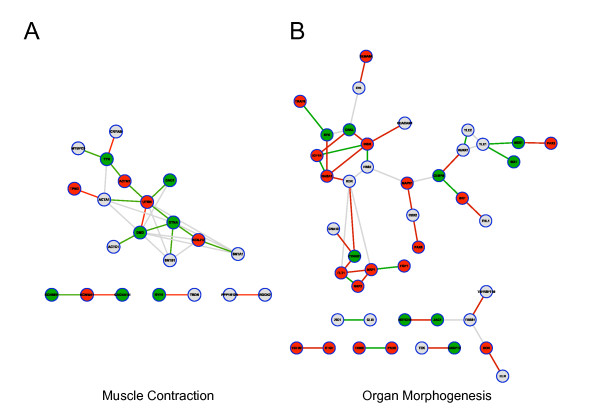
**Visualization of the identified DCM-related modules**. (A) Muscle contraction and (B) organ morphogenesis. Down-regulated SDEGs are represented by green nodes, up-regulated SDEGs by red nodes and non-SDEGs by grey nodes. Edges represent the physical interactions between proteins in HPRD PIN. Co-expressions are represented by red edges and anti-expressions by green edges.

**Figure 3:**
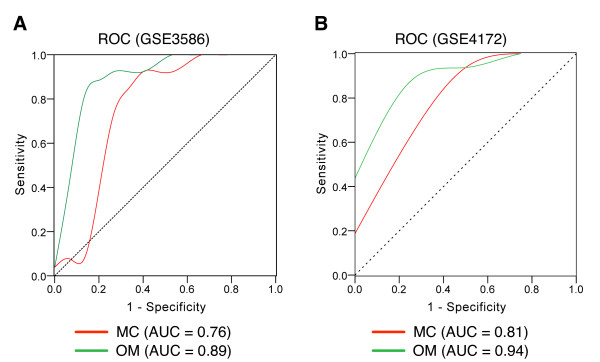
**Classification performance using the identified DCM-related modules**. ROC and AUC values were evaluated with two independent gene expression profiles: (A) GSE3586, and (B) GSE4172. OM: organ morphogenesis; MC: muscle contraction.

Based on the condition-specific CePINs, we propose a new analysis approach to identify condition-specific functional modules. First, for any specific biological condition under study, we selected significantly enriched functions among proteins in condition-specific CePIN. For each enriched function, hypergeometric tests were performed to examine whether the co-expressed interactions between two common-function proteins are also significantly enriched. By applying the proposed analysis to DCM and non-DCM CePINs, two functional modules; muscle contraction and organ morphogenesis, were specifically revealed for DCM, as shown in Figure 2. Table 4 shows the information of the network structure, hypergeometric test results, and classification accuracy of these two modules, respectively. Both modules can be used to discriminate DCM from normal tissue samples with a classification accuracy of 0.79 (muscle contraction) and 0.86 (organ morphogenesis). We further assessed their classification performance using receiver operator characteristic (ROC) curves and tested these with the expression profiles (GSE4172) from another cohort of DCM patients. The expression levels of the genes in each module were averaged to compute a module activity score. This revealed typical area under curve (AUC) values of 0.75 ~ 0.90 as shown in Figure 3. These results indicate that the identified two modules might serve as potential therapeutic targets of DCM.

**Table 4:** Summary of the two identified DCM-related modules

Module	Node	E*p*	Edge	D*p*	Accuracy	AUC
Muscle contraction	21	0.016	18	0.008	0.79	0.76
Organ morphogenesis	43	0.017	34	0.007	0.86	0.89

The list includes numbers of nodes and edges, hypergeometric test results (E*p*: *P*-value for functional enrichment; D*p*: *P*-value for functional dyad enrichment) and classification performance (accuracy and AUC).

**Figure 4:**
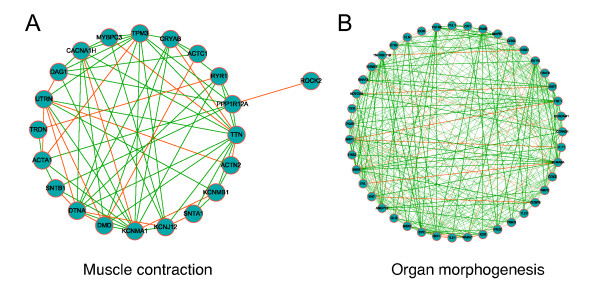
**Co-expression networks of the identified DCM-related modules**. Visualization of co-expression networks of (A) muscle contraction and (B) organ morphogenesis modules. Co-expressed protein-protein interactions are represented by red edges and other co-expressed gene pairs by green edges.

To reveal connections between member genes in both modules, their gene co-expression networks were studied and visualized in Figure 4. Both networks were organized with dense structure. The expression profiles of member genes in each module correlated more significantly with each other than those in random subnetworks of equal size (organ morphogenesis: 0.47 versus 0.36 for averaged PCC, *Z *= 5.1, *P *< 0.001; muscle contraction: 0.45 vs. 0.36, Z = 2.62, *P *= 0.06). These results suggest that strong connections within each module exist at the transcriptional level.

Subsequently we compared conditional gene expression levels and correlations of gene co-expression between DCM and non-DCM samples to illustrate the dynamic features of identified functional modules. We found that the average expression levels of member genes in each module changed between two conditions by an amount larger than expected from random subnetworks of equal size (organ morphogenesis: 0.59 vs. 0.39 for average gene expression difference, Z = 2.58, *P *= 0.01; muscle contraction: 0.58 vs. 0.39, Z = 1.7, *P *= 0.06). Moreover, the average change of member PPI gene expression correlation between conditions were also significantly higher (organ morphogenesis: 0.58 vs. 0.44, Z = 2.56, *P *= 0.01; muscle contraction: 0.56 vs. 0.44, Z = 1.75, *P *= 0.05).

**Figure 5:**
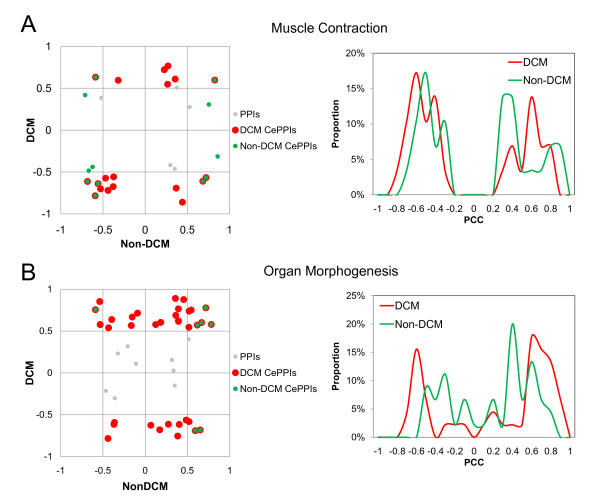
**Analysis of CePPI variations in the identified DCM-related modules**. Scatter plots (left) and histograms (right) for PCC values of PPIs in muscle contraction and organ morphogenesis modules in DCM and non-DCM respectively, are shown. In the scatter plot, each dot presents a PPI in the modules; red and green dots represent PPIs with significant correlations (PCC ≥ 0.5 or PCC ≤ -0.5) in DCM and non-DCM samples respectively; green dots with red border represent PPIs with significant correlations in both DCM and non-DCM samples. In the histograms, x-axis represents the categories of PCC and y-axis the frequency (proportion). In addition, green and red lines display the frequencies of PCC for non-DCM and DCM PPIs contained in the modules. (A) Muscle contraction. (B) Organ morphogenesis. Figure 5B suggests that the organ morphogenesis module is more activated in DCM samples, since in the scatter plot, there are more dots of CePPIs for DCM distributed on the significant area than for non-DCM and histogram of DCM also expands to two significant areas than non-DCM. On the other hand, Figure 5A suggests that the muscle contraction module is activated in both non-DCM and DCM samples, because these two distributions for non-DCM and DCM have a high degree of overlap in the histogram. However, the red dots in the scatter plot do not significantly overlap with the green dots, suggesting that these two distributions are produced by different CePPIs.

Figure 5B shows that gene pairs associated with PPIs in the organ morphogenesis module were more strongly correlated in DCM than in non-DCM, implying its activation in DCM. Meanwhile, in the muscle contraction module, gene pairs with PPIs were strongly correlated in both DCM and non-DCM (see Figure 5A, histogram), indicating that it is activated in both DCM and non-DCM. Nonetheless, the CePPIs of muscle contraction module in DCM were quite different from that in non-DCM (see Figure 5A, scatter plot), suggesting that genes in the muscle contraction module changed their associated partners between non-DCM and DCM. Although the muscle contraction module was not significantly differentially expressed and activated in both DCM and non-DCM, it was still discovered by our method due to its diverse CePPIs between DCM and non-DCM. Therefore, the muscle contraction module would have been omitted if the transcriptomic data were not integrated into the interactome data to study the condition-specific PPIs or CePPIs.

**Figure 6:**
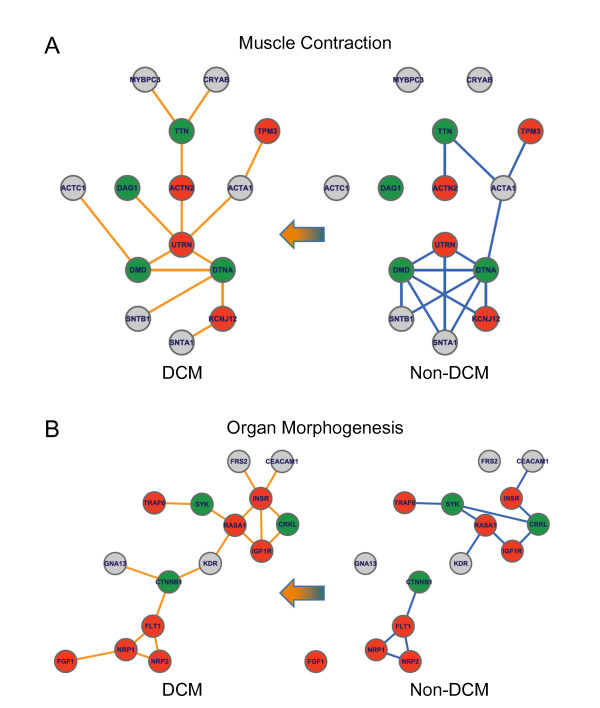
**Dynamic features of the identified DCM-related modules**. Dynamic features within these two DCM-related modules are expressed by the alternation of CePPIs in non-DCM and DCM conditions. (A) Muscle contraction. Hubs of this module are DMD and DTNA in non-DCM, but are changed to UTRN in DCM. (B) Organ morphogenesis. As the condition shifted from non-DCM to DCM, the connection is rebuilt by the interaction made from KDR and CTNNB1.

We further studied the largest connected components of these two modules with respect to the co-expressed protein interaction difference between DCM and non-DCM to determine their dynamic features. In the muscle contraction module, DTNA, SNTA1, SNTB1, and DMD were shown to be highly correlated with each other in non-DCM CePIN, but not in DCM CePIN (see Figure 6A). Proteins encoded by DTNA, SNTA1, and SNTB1 are components of the cytoplasmic part of dystrophin-associated protein complex (DAP) [14]. In addition, pivot proteins in both non-DCM and DCM, which have relatively more co-expressed interacting partners, were observed to change from DTNA and DMD to UTRN.

Similar dynamic features were also observed in the organ morphogenesis module. We roughly defined this module into two major regions: the upper diamond, formed by INSR, CRKL, IGF1R and RASA1, and the bottom triangle, by FLT1, NRP2 and NRP1 (see Figure 6B). From the results, it is evident that the communication between the diamond and the triangle in DCM CePIN was bridged by KDR and CTNNB1, but disconnected in non-DCM CePIN. Moreover, the diamond structure in non-DCM CePIN was observed to have collapsed. These changes in the muscle contraction and organ morphogenesis modules may hold some clues to the progression of DCM.

## Discussion

Protein-protein interaction networks cover all possible interactions regardless of when or where the interaction takes place. In this sense, they are static. By integrating gene expression profiles of DCM with human protein-protein interaction networks, we successfully extracted two co-expressed protein interaction networks (CePINs), i.e. DCM and non-DCM. Here, we showed that DCM and non-DCM CePINs exhibit substantial differences in co-expressed protein-protein interactions, even though their network structures are similar. The differences may be attributed to gene expression variations and interaction rewiring between DCM and non-DCM conditions. We suggest that CePINs are able to reveal condition-specific interactions and the dynamic features hidden in static protein-protein interaction networks.

Next, we showed that hub proteins in CePINs tended to be SDEGs compared to non-hub proteins. In CePINs, proteins with higher degrees imply that they have more direct interacting partners co-expressed in gene expression levels; therefore any significant modification in their expression levels might influence more interacting partners. This observation suggests that once gene expression of hub proteins is changed, it is expected to cause greater expression variations to its neighboring interaction network in DCM.

Since our analysis relies on PCC calculated from gene expression data to define CePPIs and construct condition-dependent networks, we have carried out some further examinations about its robustness. First, we performed the same analysis with tightened PCC threshold, *P *< 0.01, and obtained consistent results (see Table S1 - S4, Figure S1 - S2 in Additional File 1). Second, Li *et al*. [15] recently revealed that correlations of gene expression levels to disease states could vary a lot with randomly selected subsets of the samples from one single microarray data set. Under this light, we have performed re-sampling of our gene expression data and found that both the recovery rate of CePPIs and the identification rates of the two DCM-related modules decreased as we lowered the sample size (Figure S3 - S4 in Additional File 1). However, the identification rates of the two DCM-related modules were much higher than any other modules, indicating that these two identified modules were robustly related to DCM.

Since the human PPI data are still incomplete and noisy, there are different curated collections of human PPI data available. To examine whether our analysis was robust against the PIN we used, we performed the same analysis with an expanded PIN integrating the PPI data from HPRD [16] and BioGRID [17] databases and obtained consistent results (see Table S5 - S8, Figure S5 - S6 in Additional File 1).

One of the major symptoms of heart failure is the inability of the heart to sufficiently supply blood flow to the rest of the body. This is strongly related to heart muscle contraction efficiency. The failing heart undergoes morphological changes and becomes weakened and enlarged in DCM, the most common form of cardiomyopathy. Our findings in DCM-related modules of muscle contraction and organ morphogenesis were consistent with the known symptoms of cadiomyopathy. Consequently, we further investigated these two modules in relation to the underlying molecular mechanisms of dilated cardiomyopahty.

In the muscle contraction module, three SDEGs, DMD, DTNA and UTRN, form a closed loop, implying topological significance. Dystrophin, encoded by DMD, is a recessive, fatal X-linked disorder. It appears to stabilize the sarcolemma and protects muscle fibers from long-term contraction-induced damage and necrosis [18], though its precise roles at the cellular level are still to be elucidated. Dystrobrevin-alpha, encoded by DTNA, belongs to the dystrobrevin subfamily of the dystrophin family and is a component of the dystrophin-associated protein complex (DAP) [14]. Disruption of DAP is associated with various forms of muscular dystrophy. Dystrophin binds to the intracellular cytoskeleton by associating with actin filaments at its N-terminus, whereas at its C-terminus dystrophin interacts with members of the DAP, such as β-dystroglycan, which is encoded by DAG1. Dystrophin therefore links the intracellular microfilament network of actin to a complex series of linking proteins in the cell membrane, and hence to the extracellular matrix [19]. In the muscle contraction module, both DMD and DTNA were significantly down-regulated. The absence of dystrophin was suggested to cause the collapse of the entire DAP and plasma membrane, leading to muscle damage [14]. On the other hand, mutations of DTNA are associated with left ventricular non-compaction with congenital heart defects [20]. These defects might lead to cardiac muscle damage and possibly DCM.

**Figure 7:**
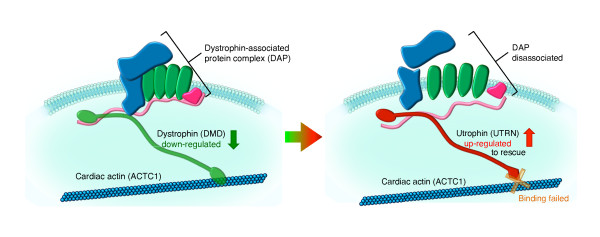
**Hypothesis of the underlying molecular mechanism of DCM**. In a normal myocardium cell, the dystrophin encoded by DMD builds a link between the intracellular microfilament network of actin and the dystrophin-associated protein complex (DAP) to protect the myocardium from long-term contraction-induced damage and necrosis. In the left panel, down-regulation of dystrophin (green rod-shaped protein) causes the collapse of the DAP and plasma membrane, leading to myocardium damage and possibly heart failure. To rescue this defect, utrophin (red rod-shaped protein) encoded by UTRN, which is the functional substitute for dystrophin, is up-regulated, as shown in the right panel. However, utrophin (UTRN) could not bind to the actin ACTC1 in cardiac cells. Hence, the connection between the actin and DAP is broken, leading to the disassociation of the DAP. As a result, cardiac cell membranes become permeable and gradually lyse, resulting in tissue destruction and heart failure.

Utrophin encoded by UTRN shares both structural and functional similarities with the dystrophin gene. Mouse studies suggested that utrophin may serve as a functional substitute for the dystrophin gene and can be viewed as a rescue protein in muscular dystrophy caused by abnormal dystrophin expression [21]. In our results, UTRN was significantly up-regulated, which might illustrate the rescue action of utrophin in the absence of dystrophin. However, no protein-protein interactions exist or are found between UTRN and ACTC1, which encodes the actin in cardiac cells, suggesting that progressive heart failure could be due to the failure of UTRN rescue. Based on these results, we proposed a hypothesis to explain how the muscle contraction module affects DCM progression (Figure 7). Recently, *Bostick *and colleagues [22] reported that utrophin up-regulation alone (without dystrophin expression) leads to DCM in mice, which provides strong evidence in support of our hypothesis.

On the other hand, in the organ morphogenesis module, we noticed that the largest connected component contains two major clusters. The two major clusters consisted of insulin pathway-related genes, including IGF1R (insulin-like growth factor 1 receptor) and INSR (insulin receptor) and vascular endothelial growth factor (VEGF) pathway-related genes, including FLT1 (VEGFR1), NRP1, NRP2 and KDR (VEGFR2). These two pathways have both been reported to be important in cardiac remodeling [23]. Proper IGF1R and INSR signaling plays an essential role in cardiac function, and the disruption of this signaling induces the onset of DCM in knockout mice [24,25]; while the VEGF pathway is crucial in vasculogenesis and angiogenesis, which was reported to be altered in DCM [26]. The significant up-regulation of these two clusters of genes can therefore signify autosomal repair for damage caused by hypoxia induced by early DCM symptoms. Malfunction of these activated pathways are possible reasons for the disease progression.

With focus on these pathways, we compared the subnetworks in DCM and non-DCM patients and found several notable points. First, these two clusters were not independent from each other, but were linked by a string of genes: RASA1, KDR, CTNNB1, and FLT1. RASA1, encoding p120-RasGAP which activates RAS GTPase, is best known for its negative regulation of the RAS/MAPK pathway downstream of several growth factor pathways responsible for cell proliferation, including the IGF-1, insulin and VEGF pathway. Proper activation of the RAS-dependent pathway is important for the functions of these pathways. The up-regulation and the linkage between RASA1 and INSR may infer a possible negative regulation of the Ras-dependent pathway. We also found CrkL, a protein that mediates Ras-dependent activation, to be significantly down-regulated in DCM patients. These observations imply the negative regulation of growth factor signaling involving insulin and insulin-like growth factor. Second, the down-regulation of CTNNB, which encodes beta-catenin in VE-cadherin essential for contact inhibition of VEGF-induced proliferation [26,27], infers a malfunction in the control of VEGF-induced vasculogenesis. The failure of beta-catenin regulation and defective vascularization have been observed in idiopathic DCM [26].

Our findings regarding the organ morphogenesis module successfully revealed possible integration of two important pathways in DCM and the crucial role that RASA1 up-regulation and CTNNB1 down-regulation might play in the etiology of DCM.

## Conclusions

Altogether we have developed a network-based comparative analysis approach that integrates protein-protein interactions with gene expression profiles and biological function annotations to reveal dynamic functional modules under different biological states. Application to DCM reveals two functional modules with dynamic features accounting for the underlying disease mechanisms. The revealed molecular modules might be used as potential drug targets and provide new directions for heart failure therapy.

## Methods

### Protein Interaction Network and Expression Data

The human protein interaction network (PIN) was downloaded from Human Protein Reference Database (HPRD) [16], and only the largest connected component, containing 9,059 proteins and 34,869 interactions, was studied.

The gene expression data of DCM were retrieved from Gene Expression Omnibus (GEO), accession number GSE3586 [8], containing 37,530 genes and 28 samples (13: DCM, 15: non-DCM) in total, with 6,475 genes involved in HPRD PIN. Another set of expression profiles of DCM was retrieved from GEO accession number GSE4172 [10] to evaluate the classification performance of identified modules.

### Co-expressed Protein Interaction Networks

**Figure 8:**
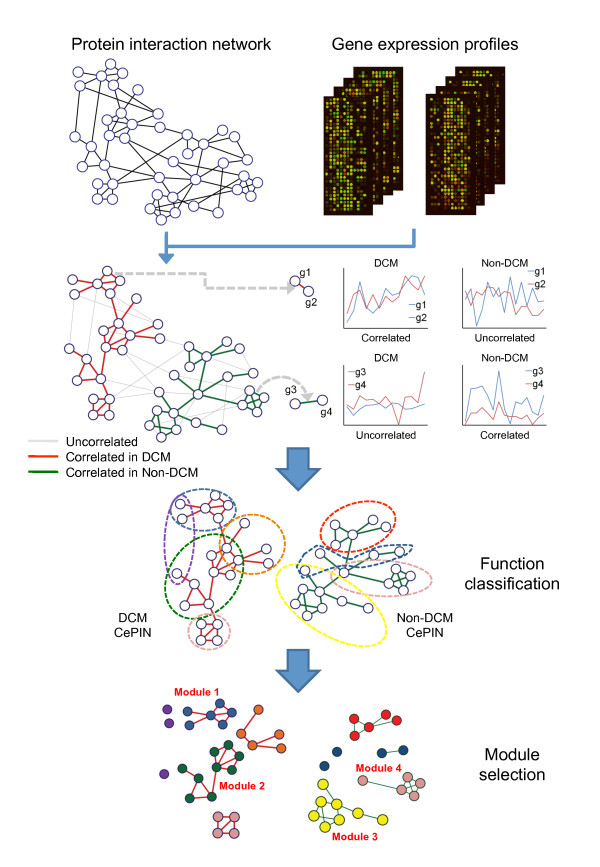
**Flow diagram of the proposed integrative network analysis procedure**. Flow diagram of discovery DCM related modules from static PIN by integrating gene expression profiles. In static PIN, each PPI was assigned a correlation derived from Pearson correlation coefficient between paired genes engaged in a PPI. Then, CePPIs and PPIs with significant correlations were utilized to construct the CePINs which were extracted from the static PIN. Next, gene sets which are provided with significant functional enrichment in GO annotation categories were grouped and isolated from CePINs. Finally, functional subnetworks which were only involved in DCM CePIN were treated as candidate DCM related modules. In addition, only candidate DCM related modules with GO annotations of levels ≥ 5 were considered as DCM related modules.

To obtain the condition-specific information, an auxiliary network, called the co-expressed protein interaction network (CePIN) was constructed. This procedure involved several data integration and search steps for extracting CePINs from HPRD PIN (or called "static" PIN as opposed to condition-specific CePIN), as outlined in Figure 8. Correlation of gene expression profiles between each pair of interacting proteins in PIN is evaluated by Pearson correlation coefficient (PCC).

PCC of paired genes (*X *and *Y*), which encode interacting proteins in PIN, is defined as:

$$PCC(X,Y)=\frac{1}{n-1}\sum_{i=1}^{n}\left(\frac{Exp(X,i)-\bar{Exp}(X)}{\sigma (X)}\right)\cdot \left(\frac{Exp(Y,i)-\bar{Exp}(Y)}{\sigma (Y)}\right),$$

where *n *is the number of condition-specific samples; *Exp*(*X*,*i*)(*Exp*(*Y*,*i*)) is the expression level of gene *X *(*Y*) in the sample *i *under a specific condition (DCM or non-DCM); $\bar{Exp}(X)$($\bar{Exp}(Y)$) represents the average expression level of gene *X *(*Y*) and *σ(X) *(*σ(Y)*) represents the standard deviation of expression level of gene *X *(*Y*). Larger absolute values of PCC indicate higher correlation between evaluated gene pairs. Those with a *P*-value of less than or equal to 0.05 were considered as significantly correlated. Protein-protein interactions between proteins encoded by significantly correlated gene pairs are defined as co-expressed protein-protein interactions (CePPIs). Based on this definition, the co-expressed protein interaction network (CePIN) is defined as the set of CePPIs. Note that we defined CePPI as co-expression in gene expression levels instead of protein concentrations since we were lack of the corresponding proteome data. Although a gene expression level cannot always represent its protein concentration, previous studies have observed notable correlations between them [28]. If proteome data are available, the same analysis procedures described here can be applied with replacement of *Exp*(*X*) and *Exp*(*Y*) by protein concentrations.

### Significantly Differentially Expressed Genes and Hub Proteins

Significantly differentially expressed genes (SDEGs) were determined by Wilcoxon rank sum test (*P *≤ 0.05, DCM against non-DCM). Up-regulated and down-regulated SDGEs were defined as genes expressed significantly higher or lower respectively in DCM than in non-DCM. DCM hub proteins were defined as nodes involved with more than 23 DCM CePPIs, since these proteins were among the top 1% of the CePPI degree distribution of the DCM CePIN.

### Topological Analysis of Networks

Four topological measures were used in this analysis: degree, betweenness centrality, closeness centrality and clustering coefficient [29]. The degree is the number of observed interactions of a given protein. Betweenness centrality (BC) measures the importance of a specific protein *i *in relating any paired proteins in PIN, and is defined by:

$$BC_{i}=\frac{SP_{i}}{C_{2}^{N}},$$

where *SP_i _*is the number of shortest path pass through protein *i *and *N *represents the number of proteins in PIN. Closeness centrality (CC) represents how close an evaluated protein is relative to all the others and is defined as the reciprocal of the mean of the shortest path lengths ($SPL_{i}$) from protein *i *to the rest of proteins in PIN,

$$CC_{i}=\frac{1}{\bar{SPL_{i}}}.$$

Higher closeness centrality implies shorter distance between the evaluated protein and all the other proteins in PIN. The clustering coefficient (*C*) of a protein determines how frequently its interacting partners interact with each other and is defined by:

$$C_{i}=\frac{e_{NB_{i}}}{C_{2}^{\left|NB_{i}\right|}},$$

in which *e_NB _*is the number of observed interactions between interacting partners of protein *i*, and |*NB_i_*| represents the number of its interacting partners. $C_{2}^{\left|NB_{i}\right|}$ gives the number of all possible interactions among its interacting partners. In this study, only the largest connected component of each CePIN was considered.

### Identification of Condition-Specific Functional Modules

To identify heart failure related modules, we applied a comparative analysis of CePINs. This analysis included several steps of functional module discovery and selection, as illustrated in Figure 8. First, GO annotation was utilized to choose gene sets, which are involved in DCM (or non-DCM) CePIN, with significant enrichment of functional categories in biological process ontology (*P *≤ 0.05). The *P*-value of functional enrichment is determined by a hypergeometric test. The hypergeometric distribution is described as follows:

$$P(X=k)=\frac{\left(\begin{array}{c} m\\ k \end{array}\right)\left(\begin{array}{c} N-m\\ n-k \end{array}\right)}{\left(\begin{array}{c} N\\ n \end{array}\right)}.$$

*X *denotes the evaluated functional category in GO. *N *represents the number of GO annotated genes, which appeared in our microarray data as well as in HPRD PIN, while *m *represents that in DCM or non-DCM CePIN. *n *represents the number of genes which are annotated as the evaluated GO functional category in HPRD PIN. Thus, this formula calculated the probability of the evaluated functional category that had k genes in CePIN. The calculated *P*-value is then adjusted by applying the Benjamini and Hochberg multiple testing procedure [30] to control the false discovery rate (FDR) at significance level of 0.05.

To further retain significantly denser functional subnetworks, we tested the functional homogeneity of each significant functionally enriched gene sets in respect to CePPIs. For this purpose, we defined functional dyads as paired proteins that formed CePPIs and shared common biological process as annotated by GO. The significance of the functional subnetwork is determined by *P*-value (≤ 0.05) derived from a modified hypergeometric test:

$$P_{e}(X=k_{e})=\frac{\left(\begin{array}{c} m_{e}\\ k_{e} \end{array}\right)\left(\begin{array}{c} N_{e}-m_{e}\\ n_{e}-k_{e} \end{array}\right)}{\left(\begin{array}{c} N_{e}\\ n_{e} \end{array}\right)},$$

*e *is the abbreviation of the functional dyad. Each symbol represents the same meaning with the previous one in the original hypergeometric test, but the counting targets are changed from functional genes to functional dyads. The same multiple testing procedure is performed to adjust the *P*-value.

After these two tests, only subnetworks with significantly high functional homogeneity in both genes and interactions would be kept for follow-up comparative analysis in order to reveal candidate DCM related modules. We defined *F*_DCM_candidate _as the set including all GO annotations of significantly denser functional subnetworks in DCM and *F*_non-DCM_candidate _in non-DCM. Then, *F*_DCM_exclusive _could be defined as the difference between *F*_non-DCM_candidate _and *F*_DCM_candidate_:

$$F_{DCM\_exclusive}=F_{DCM\_candidate}-F_{non-DCM\_candidate}$$

Functional subnetworks mapped in *F*_DCM_exclusive _will be designated as candidate DCM related modules. For functional specificity, only candidate DCM related modules with GO annotations greater than or equal to level 5 were considered as DCM related modules.

### Evaluation of Classification Accuracy

Hierarchical clustering was used to group samples into two categories (i.e. DCM or non-DCM) according to the gene expression levels of members in each module. Gene expression distance between different samples was calculated by Euclidean distance. The classification tree produced by hierarchical clustering was separated at the root into two groups (sub-trees). The group with more DCM samples is defined as positive and the other group is negative. The number of DCM samples in the positive group is defined as true positive (*TP*) and the number of non-DCM samples clustered into this group is false positive (*FP*). The number of non-DCM samples in the negative group is defined as true negative (*TN*) and the number of the rest DCM samples is false negative (*FN*). They were used to evaluate sensitivity ($\frac{TP}{TP+FN}$) and specificity ($\frac{TN}{FP+TN}$). Then, accuracy was calculated by $\frac{Sensitivity+Specificity}{Numberofsamples}$ and was used to measure the classification ability of each module. The receiver operating characteristic curve (ROC) was obtained according to the module activity score of each sample, which was defined as the average expression level of all member genes in the module.

## Authors' contributions

CCL implemented the computational method and carried out the analysis. JTH, CYW, and YJO helped to perform the analysis. CCL, JTH, and CYW drafted the manuscript. CCL and HCH conceived the study. HFJ and HCH participated in the design and coordination of the study and helped to draft the manuscript. All authors read and approved the final manuscript.

## Supplementary Material

Additional file 1 **Supplementary information**. This file contains the robustness analysis of our results in different thresholds of PCC, sample sizes of gene expression profiles, and protein interaction network. Click here for file
