# Supplementary material for: Dynamic functional modules in co-expressed protein interaction networks of dilated cardiomyopathy
Source: BMC Syst Biol. 2010 Oct 15;4:138. doi: 10.1186/1752-0509-4-138 (PMC2978157; doi:10.1186/1752-0509-4-138)
Supplement: Additional file 1 — Supplementary information. This file contains the robustness analysis of our results in different thresholds of PCC, sample sizes of gene expression profiles, and protein interaction network. [file 1752-0509-4-138-S1.pdf]

Dynamic functional modules in co-expressed protein interaction networks of dilated cardiomyopathy

Chen-Ching Lin, Jen-Tsung Hsiang, Chia-Yi Wu, Yen-Jen Oyang, Hsueh-Fen Juan* and Hsuan-Cheng Huang*

Supplementary Information

Section S1: Analysis with tightened threshold *P* < 0.01 for PCC

Section S2: Re-sampling analysis for PCC

Section S3: Analysis with integrated PIN from HPRD and BioGRID

Section S1: Analysis with *P* < 0.01 for PCC

Table S1 - Structural information of DCM and non-DCM CePINs

	Proteins	PPIs	Co-expressed PPIs
DCM	1405	5855	1199
non-DCM	1576	6663	1517
Overlap	692	3105	154

*DCM and non-DCM CePINs shared almost 50% of proteins and near 50% of physical protein interactions in common. However, the shared CePPIs were only around 10%.

Table S2 - Comparison of key topological properties

	DCM CePIN		
Property	SDEGs	Non-SDEGs	P-value
Degree	3.58	2.52	1.68E-11
Betweenness centrality	0.0013	0.0007	1.89E-10
Closeness centrality	0.2601	0.2742	5.23E-02
Clustering coefficient	0.04	0.03	3.46E-07
	Non-DCM CePIN		
Degree	4.01	2.7	4.50E-21
Betweenness centrality	0.0013	0.0006	1.71E-19
Closeness centrality	0.2527	0.2564	1.69E-05
Clustering coefficient	0.05	0.03	5.48E-14

The average of each topological property for SDEGs and non-SDEGs in either DCM or non-DCM CePIN is given in the table together with the corresponding P-value. The P-values were calculated using non-parametric Wilcoxon rank-sum tests. In both CePINs, degree and betweenness centrality of SDEGs are significantly higher than those of Non-SDEGs.

Table S3 - Top 10 significant level-6 GO annotations of hubs

GO term	Description	*P*-value
16310	**phosphorylation**	1.70E-07
6468	**protein amino acid phosphorylation**	1.49E-06
43687	post-translational protein modification	3.05E-06
18212	peptidyl-tyrosine modification	9.67E-06
7169	transmembrane receptor protein tyrosine kinase signaling pathway	1.91E-05
8629	**induction of apoptosis by intracellular signals**	3.42E-05
51173	positive regulation of nitrogen compound metabolic process	1.19E-04
7172	signal complex assembly	1.58E-04
45428	regulation of nitric oxide biosynthetic process	1.58E-04
51345	positive regulation of hydrolase activity	2.94E-04

Table S4 - Summary of the two identified DCM-related modules

Module	Node	E*p*	Edge	D*p*	Accuracy	AUC
Muscle contraction	12	< 0.01	7	0.02	0.68	0.83
Organ morphogenesis	21	< 0.01	13	0.01	0.75	0.77

Two identified DCM-related modules are also significant and perform well classification between DCM and non-DCM samples.

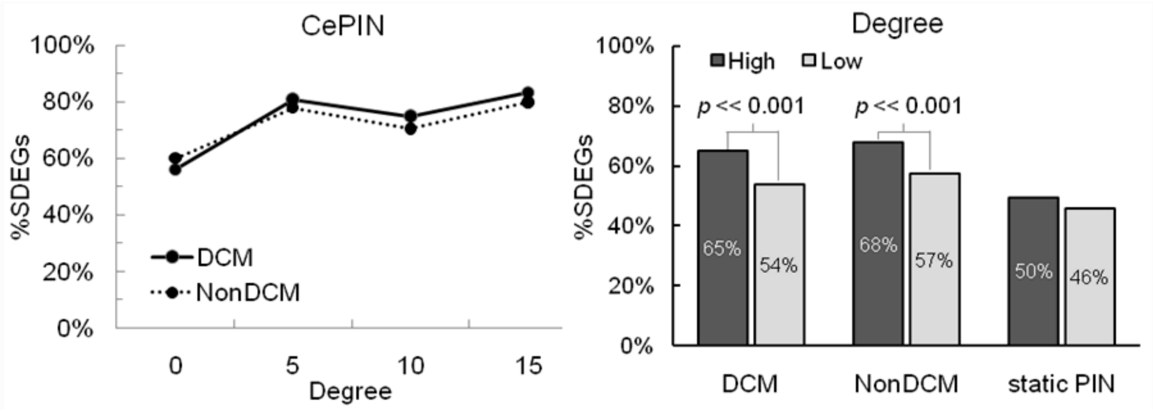

Figure S1 - Correlations between CePPI degree and SDEG proportion

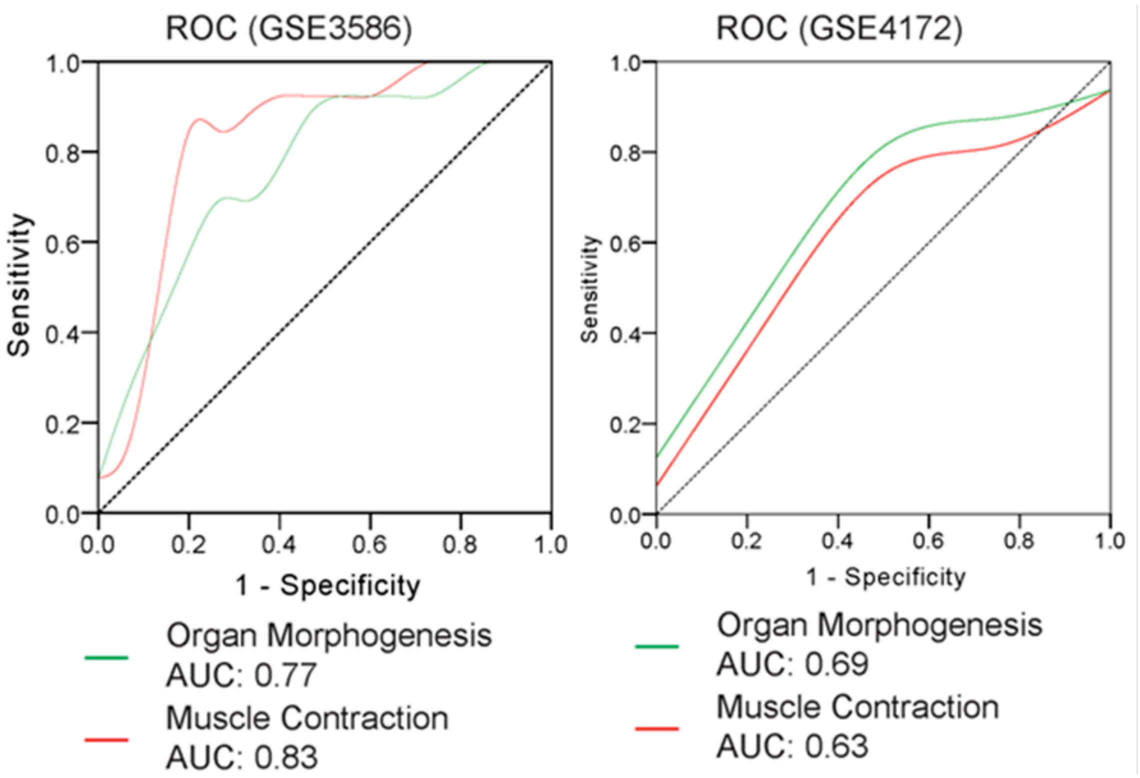

Figure S2 - Classification performance of the identified DCM-related modules

Section S2: Re-sampling analysis for PCC

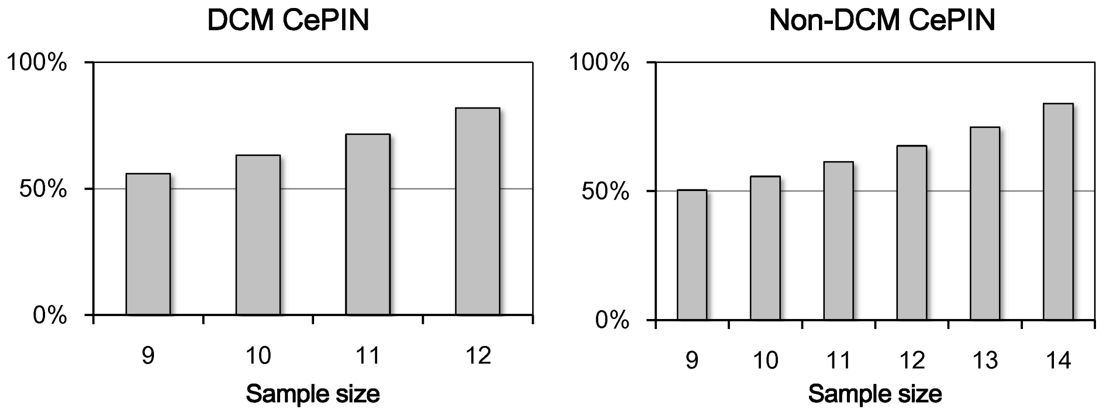

Figure S3 – Recovery rate of CePPIs

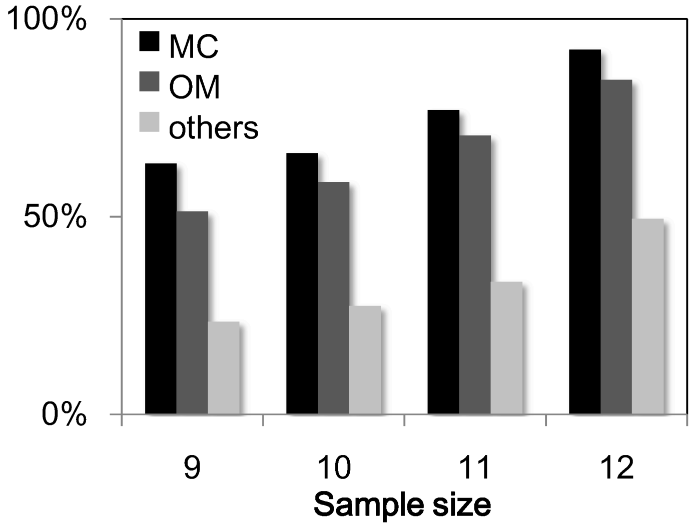

Figure S4 – Identification rate of the DCM-related modules

The identification rates of 2 DCM-related modules are obviously higher than other modules. MC: muscle contraction; OM: organ morphogenesis; others: modules which are identified during the re-sampling procedure.

Section S3: Analysis with integrated PIN from HPRD and BioGRID

Table S5 - Structural information of DCM and non-DCM CePINs

CePIN	Proteins	PPIs	Co-expressed PPIs
DCM	3494	19088	5391
non-DCM	3651	19872	6224
Overlap	2608	15882	1811

*DCM and non-DCM CePINs shared almost 70% of proteins and near 80% of physical protein interactions in common. However, the shared CePPIs were only around 30%.

Table S6 - Comparison of key topological properties

Property	DCM CePIN		
	SDEGs	Non-SDEGs	P-value
Degree	3.58	2.52	1.68E-11
Betweenness centrality	0.0013	0.0007	1.89E-10
Closeness centrality	0.2601	0.2742	5.23E-02
Clustering coefficient	0.04	0.03	3.46E-07
	Non-DCM CePIN		
Degree	4.01	2.7	4.50E-21
Betweenness centrality	0.0013	0.0006	1.71E-19
Closeness centrality	0.2527	0.2564	1.69E-05
Clustering coefficient	0.05	0.03	5.48E-14

The average of each topological property for SDEGs and non-SDEGs in either DCM or non-DCM CePIN is given in the table together with the corresponding P-value. The P-values were calculated using non-parametric Wilcoxon rank-sum tests. In both CePINs, degree and betweenness centrality of SDEGs are significantly higher than those of Non-SDEGs.

Table S7 - Top 10 significant level-6 GO annotations of hubs

GO term	Description	*P*-value
43687	post-translational protein modification	1.20E-08
31325	positive regulation of cellular metabolic process	5.21E-07
16310	**phosphorylation**	5.84E-07
6468	**protein amino acid phosphorylation**	1.64E-06
42981	**regulation of apoptosis**	2.02E-06
43067	**regulation of programmed cell death**	2.27E-06
7179	transforming growth factor beta receptor signaling pathway	1.08E-05
9891	positive regulation of biosynthetic process	1.13E-05
8629	induction of apoptosis by intracellular signals	1.73E-05
7169	transmembrane receptor protein tyrosine kinase signaling pathway	1.75E-05

Table S8 - Summary of the two identified DCM-related modules

Module	Node	E*p*	Edge	D*p*	Accuracy	AUC
Muscle contraction	24	0.01	20	0.01	0.71	0.69
Organ morphogenesis	58	0.01	46	< 0.01	0.82	0.91

Two identified DCM-related modules are also significant and perform well classification between DCM and non-DCM samples.

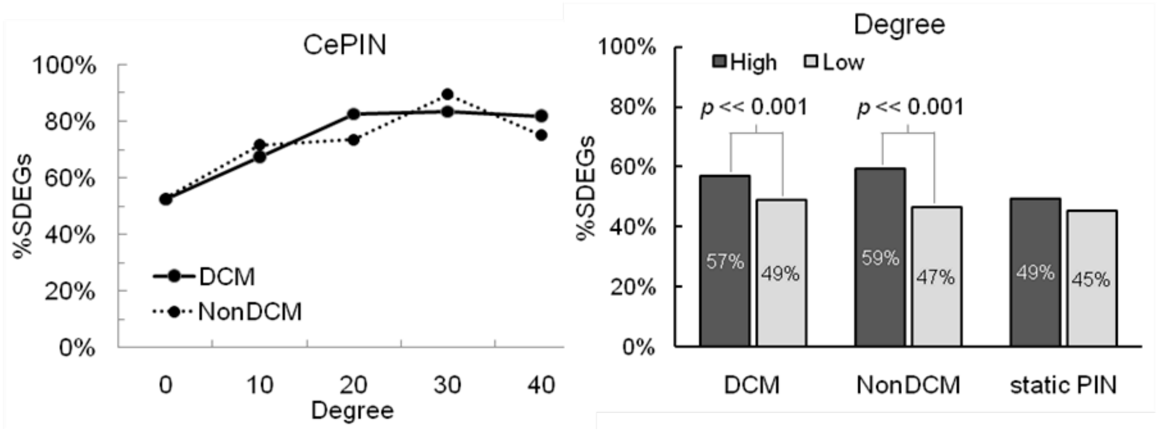

Figure S5 - Correlations between CePPI degree and SDEG proportion

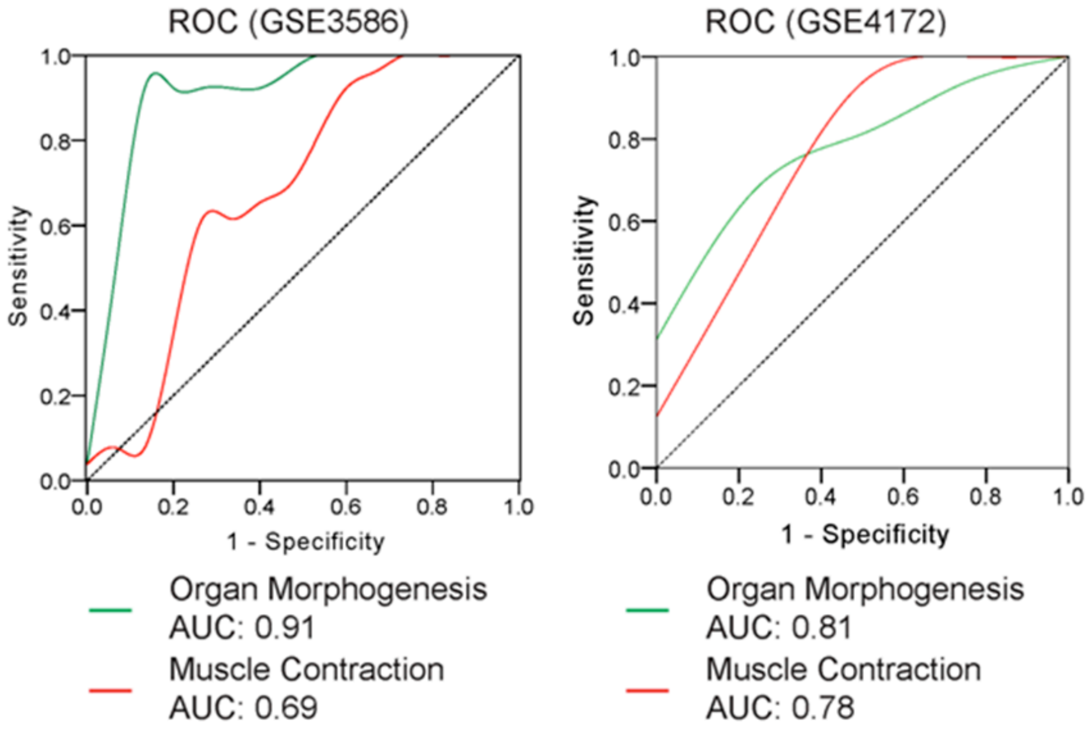

Figure S6 - Classification performance of the identified DCM-related modules
